# *“They say to me ‘You want to be a high shot and be like a tourist’ so I stopped wearing shoes at home even though I know it is to protect my feet”*. The perceptions of patients on foot complications

**DOI:** 10.1371/journal.pone.0294518

**Published:** 2023-11-17

**Authors:** Malakai Sovaki Ranuve, Masoud Mohammadnezhad

**Affiliations:** 1 Fiji Ministry of Ministry of Health and Medical Services, Rotuma Hospital, Fiji; 2 School of Nursing and Healthcare Leadership, University of Bradford, Bradford, United Kingdom; 3 Department of Health Education and Behavioral Sciences, Faculty of Public Health, Mahidol University, Nakhon Pathom, Thailand; Muhimbili University of Health and Allied Sciences School of Medicine, UNITED REPUBLIC OF TANZANIA

## Abstract

**Background:**

Diabetic Foot Ulceration (DFU) is one of the serious complications for people with diabetes and it is also the most devastating but yet preventable complication. This study aimed to explore the perceptions of Type 2 Diabetes Mellitus (T2DM) patients on their foot complications and foot care practices in Fiji.

**Methods:**

A qualitative study design was used to recruit T2DM patients attending Diabetic clinic in Rotuma Hospital, Fiji from July to September, 2021. Purposive sampling was used to recruit 27 patients until data saturation is happened. Semi-structured one-on-one in-depth interviews that were audio-recorded were used to collect data which was then transcribed and manually analyzed using thematic analysis method.

**Results:**

This study found four main themes namely Knowledge and its determinants, Perceptions on personal practice and health care practice, Health seeking behaviors and Factors affecting footwear practices. T2DM patients had varying levels of knowledge about DFU and these levels of knowledge were attributed to lack of advice from healthcare providers, personal beliefs, culture and societal norms and lack of resources. Patients continued to practice self-care practices that they perceived to protect their feet from trauma and such practices have been passed down through a traditional knowledge system including stigma and beliefs. Culture and personal habits greatly impacted the practice of wearing footwear. Societal norms and traditions greatly impact how T2DM patients take care of their feet and their health in general.

**Conclusion:**

Understanding personal beliefs and traditional influences surrounding the patients’ environment is paramount in order to effectively implement public health prevention strategies against DFU.

## Introduction

Diabetes Mellitus (DM) is a disease of chronic nature that occurs as a result of either the human body’s inability to produce enough *insulin hormone* or the human body is unable to use the insulin produced by the pancreas [1, 2]. The recommended normal value by the World Health Organization (WHO) for Random Blood Sugar (RBS) level is less than 6.5mmol/L and for Fasting Blood Sugar (FBS) is less than 5.6mmol/L. When the value for FBS is between 5.6–6.9mmol/L pre-diabetes is happened and when it is 7mmol/L or higher on two separate tests, diabetes is diagnosed [3]. DM has made its mark in the global stage by becoming a very important public health problem because its prevalence has been consistently rising over the past decades [2, 4].

The WHO estimated that over 90% of DM cases are Type 2 Diabetes Mellitus (T2DM) and have predicted a worrying trend for T2DM in the future [5]. It is also one of the leading causes of morbidity and mortality. The International Diabetes Foundation (IDF) estimated that in 2019, 4.2 million deaths were due to DM and cost a financial burden of 720 billion USD in health expenditure alone [2, 6]. The true burden of disease of T2DM may be underrepresented globally as many people remain undiagnosed, as many as 1in 3 which sums up to approximately 232 million people worldwide. The challenging aspect of T2DM is that more than 80% of people living with the disease are from Low to Middle Income Countries (LMICs) which makes it challenging to effectively control the disease and control its complications [4, 5].

The prevalence of micro-vascular complications varies from country to country and is generally estimated globally that at least 1/5^th^ of T2DM have developed some micro-vascular complications. The commonest of all micro-vascular complications include retinopathy, nephropathy and neuropathy with neuropathy as the main causative factor for the development of Diabetic Foot Ulcer (DFU) and eventually Lower Limb Amputations (LLA) [7]. Major risk factors which lead to DFU include reduced perfusion and sensation to the feet from peripheral neuropathy and peripheral arterial disease [8]. They are associated with repetitive stresses like shear and pressure on the diabetic-complicated foot and healing is delayed and complicated by infection [9]. It has also been reported that diabetic neuropathy is the most common denominator in almost 90% of DFU cases coupled with trauma and Peripheral Arterial Disease (PAD) [10].

People with T2DM are at risk of developing foot complications with a lifetime risk of 25% and somewhere around the world; someone is losing his or her lower limb simply because of diabetes foot complications. It has also been shown in one study that every 30 seconds, a limb is being amputated due to foot complications of diabetes [11]. The rise in the prevalence of diabetes and long-life expectancy in diabetic patients due to improved management and care, has led to an increase in the incidences of diabetic foot complications [12, 13].

The five year mortality rate of DFU that will eventually require LLA is the same as the most aggressive types of cancer, ranging from 40–80% [14–16]. Recurrence of DFU in those patients with already healed ulcers is common with a cumulative rate of recurrence within 5 years at 66% and 12% for amputations [9, 17].

Diabetes Fiji has reported that the rate of amputations in Fiji is one in every 12 hours and this staggering rate is worsened by contributing factors such as poor sanitation and hygienic living conditions, practice of walking barefoot, injuries and rodent bites which are all common amongst Fijians [18]. Data from the Diabetic Foot Clinic in Rotuma Hospital showed 12 cases of diabetic patients attending diabetic foot clinic for active diabetic foot ulcers, and 5 cases were referred to tertiary hospital for surgical intervention and amputation for the financial year 2019–2020 [19]. The health seeking behaviour of Fijians is deeply embedded into their culture and their environment and is worsened by the fact that there is a lack of clinical expertise in podiatry in Fiji [18, 19].

An estimation of 75% of DFU can easily be prevented by quality standard preventive methods. It has also been noted that the cornerstone to the prevention of these diabetic foot complications is patient’s engagement in foot care. Their perspectives in the promotion of engagement in foot care is vital as studies have shown that existing interventions are ineffective as it does not consider the behaviour and perceptions of patients on diabetic foot care disease [17, 20].

No studies have been conducted on the island of Rotuma, Fiji to explore the perceptions of T2DM patients on foot complications and foot care practices. There are some questions that this study is aiming to answer such as What are the perceptions of T2DM patients regarding diabetic foot care and foot complications? What are some barriers that T2DM patients face in practicing foot care? and What are some possible solutions to promoting foot care practices among T2DM patients? Hence, this study was conducted with the aim of exploring the perceptions and beliefs about diabetic foot complications and foot care practices amongst T2DM patients attending the Diabetic Clinic at Rotuma Hospital.

## Methodology

### Study design and setting

This study was a qualitative study which employs a method of study to explore opinions, views, thoughts, feelings, knowledge and experiences of T2DM patients. The study was conducted at Rotuma subdivisional hospital, Eastern Division, Fiji, from July to September, 2021. Rotuma, with a population of approximately 2,000 people, is part of Fiji and has only one Primary Health Care (PHC) hospital delivering health care services to the people of Rotuma. The study was conducted amongst T2DM patients attending the Diabetic Clinic at Rotuma Hospital Special Outpatients Department (SOPD). All T2DM cases attend this clinic for general management of their diabetes as well as end-organ damage assessment which includes foot care services.

### Study sample

The study sample included all T2DM patients registered and attending in the Diabetic Clinic at Rotuma Hospital Special Outpatients Department (SOPD). Those selected included anyone with T2DM and attending the Diabetic Clinic at Rotuma Hospital SOPD for more than a year, male or female, must be more than 18 years old, identified as a Fijian or Rotuman and must be living in Rotuma for at least a year. Those with other types of DM, those with any medical conditions that were not able to participate in this study and those who did not consent were excluded from the study.

T2DM patients were purposively selected and this was done to recruit participants who were able to provide in-depth and detailed information about their perceptions regarding diabetic foot complications and foot care in the Rotuma Hospital. Sampling continued until data saturation was reached hence 27 were included in the study.

### Data collection tool

Data was collected through the one-on-one in-depth interview sessions using semi-structured interview questionnaires. This method was used since it is one of the most effective ways of qualitative data collection methods as it involved direct engagement with each individual participant. Additionally, patients were not comfortable but instead shy talking about their personal health in front of a group of people hence one-on-one interviews was more appropriate and effective in collecting data. It also allows interviewers to ask appropriate follow-up questions, probe deeper and may later make another round at the key questions to get a better, richer perspective of the participants on the issue of interest [21].

The questionnaire was developed in two versions, one in the English language and the second one in the Rotuman language. Pilot tests were conducted to test the two versions of the questionnaire. Necessary changes were made before the final questionnaires were used as an interview guide in the data collection (Table 1).

**Table 1:** Interview questionnaire for T2DM patients.

1	Do you remember the year you were first diagnosed with T2DM?
2	What do you understand about diabetes?
3	What do you know about complications of T2DM?
4	Do you know what foot complications are? Please explain?
5	What do you think is the main cause of DFU in T2DM?
6	Has any HCW in Rotuma hospital talked to you about T2DM?
7	Has any HCW in Rotuma hospital talked to you about foot care?
8	Where did you first learn or hear about foot complications?
9	Please explain some of the things that make it difficult for you to take care of your feet?

### Study procedure

The recruitment of study participants was conducted by a health officer who was not involved in the care of T2DM patients attending Diabetic Clinics of Rotuma Hospital SOPD. T2DM patients were approached during their scheduled clinic times which is usually on Thursdays and Fridays of every week. T2DM patients were approached while waiting for their DM clinics and were offered participant information sheets as well as verbal explanations about the study. Those who showed interest and had met the inclusion criteria were given consent forms to sign. Participants were interviewed during their scheduled SOPD clinic times to avoid unnecessary costs of coming back to the hospital. They were escorted one-by-one to a quiet room within the hospital where face-to-face in-depth interviews were conducted. The recruitment of participants and interview sessions were conducted by a trained counsellor with experience in motivational interview technique. Those participants not comfortable in speaking in the English language were interviewed in the Rotuman language by a bilingual staff member. The sessions were audio-recorded and each interview lasting for about 30 to 45 minutes.

### Data management and analysis

The interview sessions were recorded using a voice recorder. Transcripts of the sessions were produced after every interview session; little and often and this was done to avoid piling up of recordings towards the end of the study. The recordings from the interviews conducted in the Rotuman language were transcribed in Rotuman before being translated into the English language. The English voice recordings from the one-on-one interview sessions were transcribed by the primary investigator and the assistant who conducted the interviews and compiled them for analysis. After the voice recordings had been transcribed, translated and verified that they were true representations of what transpired during the interview sessions, the data was then analyzed using thematic analysis [22, 23]. Data was analyzed concurrently as the data collection progressed. This was an effective method as it could include new ideas and questions highlighted in the process of analysis or the research question was further developed as more light was shed into the perspectives of the participants.

### Study rigor

To ensure that this qualitative study was done with extreme rigor, the following four criterion was used: credibility, transferability, dependability and confirmability [24–26]. Credibility was ensured through feedback and advice from the research team and was taken into account with the study edited accordingly. Additionally, data was collected using semi-structured interview questionnaires that were first piloted through role play sessions prior to being used in the main study. Transferability was ensured through comprehensive descriptions of the study background, significance of study and the study design. Participants were provided with the study method allowing for the reader to determine if it can be applied to the setting here. Dependability was enhanced through a detailed description of the study methods. Lastly, confirmability was ensured through peer reviews and comments regarding the study which was acknowledged with necessary edits by researchers carried out in the study report.

### Ethical considerations

Ethics approval was granted from the College Health Research Ethics Committee (CHREC) of Fiji National University (FNU) with ID#002.21 followed by facility approval from the Divisional Medical Officer, Eastern Health Services, Fiji Islands. All diabetic participants were offered consent forms and were interviewed once they had given their written consent.

## Results

### Demographic characteristics of participants

A total of 27 participants were part of the study, out of which 17 were females and 10 were males with ages ranging from 37 to more than 60 years old. The majority of participants were Rotumans with more than half being married. All participants had had some formal education; primary and secondary education with a few reaching-up to tertiary levels (Table 2).

**Table 2:** Demographic details of T2DM patients (n = 27).

Variables	Number	Percentage
**Gender**		
Male	10	37
Female	17	63
**Age (Years)**		
35–45	5	18.5
46–55	8	29.6
56 –>60	14	51.9
**Ethnicities**		
i-Taukei	2	7.4
Rotuman	25	92.6
**Marital status**		
Single	8	29.6
Married	17	62.9
Widowed	2	7.4
**Educational background**		
Primary	10	37.0
Secondary	15	55.6
Tertiary	2	7.4

### Themes identified

The one-on-one interviews with the T2DM patients generated four themes including Knowledge and its determinants; Personal practice and health care practice; Health seeking behavior and Factors affecting footwear practices; and 13 sub-themes as shown below in Table 3. The participants were coded P1 as participant 1, P2 as participant 2 and so forth.

**Table 3:** Themes and sub-themes identified.

Themes	Sub-themes
**Knowledge and its determinants**	DM knowledge
	DFU knowledge
	Foot care knowledge
	Determinants of knowledge
**Personal practice and health care practice**	Fatalistic practices
	SOPD Clinics
	Foot care clinics
	Beneficial self-care practices
**Health-seeking behaviour**	Hospital factors
	Culture, personal beliefs and traditions
**Factors affecting footwear practices**	Availability and affordability
	Personal habits, societal norms and traditions
	Access to knowledge about appropriate footwear

#### Theme 1: Knowledge and its determinants

Under this theme, the different levels of knowledge of the participants are presented based on the data collected. The theme generated 4 sub-themes namely: DM knowledge, DFU knowledge, Foot care knowledge and Determinants of knowledge. Overall, the participants have a lot of gaps in their knowledge levels.

*DM knowledge*. There were different levels and types of knowledge regarding DM and its aspects that the participants have. Five of the participants managed to explain and relate DM to high levels of glucose in the blood. One of the participants explained that it is when the sugar is high indicating that insulin is not enough to breakdown the sugar at cellular level.

“*It’s a very complicated disease…*..*it eats you up from inside both mentally and physically*. *Diabetes is when the sugar is very high when tested and it indicates a high glucose level in the blood which means that the insulin is not enough to break down the sugar that comes into your blood…”*. P12 (37 year old male)

The other four participants all articulated the same concept that it is when the sugar is high.

“*…diabetes is when our sugar goes up…”*. P17 (64 year old female)

Furthermore, another participant stated that once her sugar level was noted as being high, she was referred to the doctor and given advice.

“*Diabetes is what I know to be when the sugar is high in the blood…you come and have a check-up and when the nurse take your sugar test and the will see the number and if it is not normal*, *then they will say that my sugar is high and I can have diabetes so they refer me to the doctor to be seen regarding my high sugars”* P20 (60 year old female)

Another 4 participants shared their perception of DM as a dangerous disease that must not be taken lightly and to which every piece of advice from HCW must be adhered.

“*It’s a dangerous sickness*, *diabetes is a disease we should not take easy on it and we must be concerned with our eating habits*, *our weight and our tablets we must take on time every day”*. P16 (40 year old female)

Similarly, another participant said that it is dangerous because it can kill people and further explains that lifestyle modifications must be done prior to the administration of medications.

“*It is a dangerous disease because it can kill lives*. *Some people lose hope when they are told that they have diabetes and they depend too much on the HCWs and the medicine from the hospital*, *they can’t control their lifestyle*, *eating habits an exercise their body”*. P21 (50 year old female)

Additionally, another participant said that there are some symptoms that can be felt when the sugar is high.

“*Diabetes is a very dangerous disease because when your sugar is going up*, *you can feel it in your body like the headache and dizziness…”*. P23 (71 year old female)

DM was also perceived as a killer since people have died because of it.

“..*It is a killer and a lot of people have died from the disease”*. P26 (58 year old female)

Only one of the participants stated that DM is a Non-communicable Disease (NCD).

“*…it is a NCD and it’s not a good disease to have…”*. P22 (64 year old female)

Two participants however, mentioned that they know nothing about diabetes at all.

“*…no I don’t know what diabetes is…”*. P14 (35 year old male)

The second participant also mentioned the same thing.

“*I don’t know anything about diabetes…”*. P15 (63 year old female)

All participants described some risk factors of DM with all mentioning that unhealthy eating habits are the main culprits in the causal pathway for DM. Consumption of an unhealthy diet was the dominant risk for the development of DM perceived by the participants.

“*…it is because of eating a lot of unhealthy food*.*…foods like ice cream*, *cakes and a lot of cassava and flour*, *especially here in Rotuma we eat a lot of rice and flour”*. P10 (57 year old female)

Another participant added that too much sugar and salt on our food can lead to DM.

“*…sometimes we eat too much sugar and salty foods…yeah the can cause diabetes”*. P11 (78 year old female)

Other risk factors mentioned by the participants also included lack of exercises (three participants), genetic factors (two participants) and smoking (one participant). One participant explained that lack of exercise can contribute to DM.

“*…lack of exercises*, *not moving and riding all the time on the motor bike even for short distances can cause diabetes…”* P12 (37 year old male)

Another participant explained that it has a genetic predisposition with positive family history of DM.

“*…diabetes is also caused by the blood…like if it is in the family then we can also get it because if our mother or father ah it*, *the children can also have high risk of getting diabetes…”*. P16 (40 year old female)

Another participant added that smoking is also high-risk behaviour for the development of DM.

“*Smoking is also a risk factor for diabetes and if we smoke*, *we must try to cut down or eventually quit the smoking because it is not good for our health and smoking can damage my blood vessels and make my diabetes worse”*. P14 (35 year old male)

There was a good level of understanding regarding what the participants perceived that needed to be done to control and manage diabetes. All participants explained diet as the main way of managing their diabetes followed by exercise (two participants) and medications (two participants).

“..*If we want to control our diabetes*, *we must control what we put in our mouth…the food must be healthy food and also how it is prepared is important…must boil all the food and cut down on sugar*, *salt and red meat”*. P10 (57 year old female)

Another participant said that in addition to diet and exercise control, compliance to medications from the hospital is equally important.

“*… we must be very careful with what we eat and follow the advice from the hospital*, *exercise regularly and also we must take our tablets everyday as advised by the doctor and the nurse so that our diabetes is well controlled”*. P16 (40 year old female)

Moreover, one of the participants mentioned how the doctor explained to her the mechanism of actions for the medications that she was taking to control her DM and expressed how grateful she was for realizing that the medications are really important in helping to control blood glucose levels.

“*The doctor had explained to me one day what the medications am taking do to my body to control my sugars and it was the first time for me to hear it*, *I was very happy and now I know that medications and all these pills am taking is helping my body to control ad manage my diabetes”*. P17 (64 year old female)

With regards to the complications of DM, all of the participants managed to name some complications except for one participant who said:

“*…I am sorry but I do not know any complications of diabetes…”*. P14 (35 year old male)

For the vast majority who managed to explain the complications, liver failure, kidney failure, eye problems, DFU, stroke and heart attack were mentioned as complications of DM.

“..*If not controlled*, *diabetes can damage all the organs in our body*. *Liver*, *kidneys*, *eyes…”*. P10 (57 year old female)

Another participant stated eye problems, kidney injury and foot ulcers as complications that she read from a chart in the SOPD clinic.

“*I am not really sure what they are*, *but I have seen some pictures on the charts in SOPD clinic about the eyes*, *the kidneys and the feet with one big cut on it*, *maybe those things can happen to us if we don’t control our diabetes”*. P11 (78 year old female)

Another participant added numbness to the legs and feet to the list of complications mentioned by other participants.

“*It can lead to blindness*, *kidney failure*, *numbness in the legs and feet”*. P12 (37 year old male)

Additionally, another participant also added stroke and heart attack as complications of DM.

“*…it can lead to stroke*, *heart attack*, *blindness and all this damage to the body”*. P16 (40 year old female)

*DFU knowledge*. Knowledge on DFU was mixed amongst the participants. Seven participants expressed that they do not know anything about DFU.

“*No I don’t know anything about foot ulcers or foot complications*, *I don’t know how it happens”*. P14 (35 year old male)

Another participant mentioned that she has seen some people with amputations but how it happens is still a mystery to her.

“*Am not really sure what they are*, *I have seen some people with their legs being cut and that’s all… the cause of diabetic foot ulcers… I don’t know to be honest”*. P10 (57 year old female)

One of the participant highlighted that she is aware of how to control her sugar levels but is not aware of what DFU is.

“*All I know is controlling your food and not eating sugary foods*, *DFU*? *No idea”*. P16 (40 year old female)

On the other hand, 10 of the participants did mention that they understood and know about foot complications. Uncontrolled DM can lead to damages on the blood vessels inside the body which leads to delayed wound healing when there is a cut on the foot causing DFU.

“*…uncontrolled diabetes can cause your blood vessels to be damaged and if there is a cut on your foot*, *you may not be able to feel it and take time to heal and this is mainly because of the food we eat that give a lot of glucose in the blood”*. P12 (37 year old male)

Another participant added that with uncontrolled sugars, one small cut on the foot can progress and become worse.

“*…the feet can be cut if sugar is not controlled especially from one small cut and it can become very bad and if I get a cut or small scratches or scabies on my leg and feet*, *it can get long to heal and can spread all over my feet and can be cut off if the infection has spread*. *And this is all because of high sugars”*. P13 (58 year old female)

*Diabetic foot care knowledge*. All participants showed different levels of understanding, what they know (four participants) and don’t know (13 participants) about foot care. For the four participants who knew about foot care, one mentioned washing of their feet, one mentioned footwear while two mentioned both as part of foot care.

“*…I was told to be careful of my feet*, *wear shoes all the time and wash my feet properly with soap and water whenever I take a bath”*. P10 (57 year old female)

One of the participants stated that wearing shoes is a must to protect the feet and must be oiled twice a day.

“*All I know is that I must wear shoes all the time*, *put oil on my feet every morning and afternoon”*. P15 (63 year old female)

Another participant explained that shoes must be worn inside and outside of the house and any small cut or laceration to the feet must immediately be taken to the hospital for assessment and treatment.

“*I was advised about foot wear and to wear them everywhere*, *inside and outside the house so that I take care of my feet and make sure I don’t get any cuts on the feet*, *but if I do get some cuts or injuries*, *I must treat it properly and bring it to hospital for treatment or it may get worse and can get cut off*.*”*. P17 (64 year old female)

Additionally, one participant explained the specifics of washing the feet.

“*Always clean and wash your feet with soap and water*, *dry it nicely and apply the oil but no oil in between the toes”*. P18 (47 year old female)

On the other hand, 13 participants said that they could not remember anything about foot care. One of them said that he has never been advised about foot care from the hospital.

“*I don’t know anything about foot care as no one in the hospital has ever advised me on foot care…”*. P14 (35 year old male)

Another participant added that he has not been advised on foot care so he doesn’t know anything about it.

“*Every time I come to hospital*, *all I am advised on is diet and control your sugar; no one has told me anything about foot care”*. P16 (40 year old female)

Another participant mentioned not knowing the specifics of how to wash and clean her feet.

“*…I mean I always wash my feet whenever I have my bath but the specifics of how to take care of my feet–no idea”*. P19 (63 year old female)

Another participant similarly said that she washes her feet when she takes her bath but is not familiar with foot care.

“*…am sorry but I forgot what foot care is*, *I do wash my feet when I bath but am not sure”*. P25 (61 year old female)

*Determinants of knowledge*. Participants were also probed on what could be the reasons for the varying levels of knowledge among them. The main factors that were mentioned by the participants to have influenced how much they know regarding DM, DFU and foot care are traditional knowledge and too much or too little information from HCWs.

On traditional knowledge, participants highlighted what they heard from the older generations in the village or from relatives and friends at home has influenced the knowledge they have.

“*…I heard from some older ladies in the village that poor control of diabetes can cause me to be very sick all the time and all sickness that comes will affect me”*. P16 (40 year old female)

Another participant mentioned that he first heard about it from his uncle who had an amputation because of DM.

“*…with foot complications*, *I didn’t hear it from the hospital at first*. *I saw my uncle who had an amputation on his left leg and I asked him what happened and he told me that he had a small cut on his left foot but when he came to hospital*, *he was told that his sugar was high and so they have to send him to Suva to cut his leg because it is hard for them to treat here in Rotuma*. *From there*, *I know that cuts on the foot can damage the leg if the sugar is high”*. P12 (37 year old male)

Another participant explained that she heard it from her grandparents that this disease was brought by foreigners who came from outside Rotuma.

“*…I knew about diabetes from my grandparents before I hear it from the nurse*. *My grandparents once told me that diabetes came from the outsiders who came to Rotuma and brought all these diseases with them to us”*. P13 (58 year old male)

In addition, another participant also shared that according to her grandparents, chronic wounds on the feet that are slow to heal is because of some traditional reasons whereby those affected must have stepped on sacred sites or burial grounds.

“*My grandmother used to tell us that those with sores and ulcers on their legs that are difficult to heal and end up in the hospital to be cut off have stepped on the graves or sacred site in the village and hurt their foot*, *that’s why they have those ulcers on their legs”*. P26 (58 year old female)

With the information that participants acquire from professional HCWs, all participants highlighted that HCWs provide too much information on CBG levels and how to control them but hardly any advice on foot complications or foot care.

*“Yes the HCWs talked to me about diabetes but…no one has talked to me about complications of diabetes or foot care…”*. P11 (78 year old female)

Another participant shared the same thing as quoted above.

“*…the nurse advised me on my sugar levels that it was high*, *she also told me about what to do and how to keep myself healthy and control my sugar level but no advice on foot care or how to look after my feet”*. P10 (57 year old female)

Additionally, another participant said that all she knows is diet control because that is the main advice she gets whenever she comes in for her SOPD clinic.

“*…the main advice I get every time I visit the hospital is always on diet*, *I have never received or been advised on foot complications and foot care so when you asked me about diabetes and all this*, *all I know is eat the right kind of food in right amount… to be honest*, *no one in the hospital has talked to me about foot care”*. P16 (40 year old female)

Another participant also stated that it is always about sugar levels and diet; nothing on foot care.

“*I am always advised on my sugar levels if they are high or ok and from there*, *the nurse advise me on diet and what to do to control my sugar level and that was it”*. P24 (46 year old female)

Moreover, two participants mentioned that they were given too much information in one day that they went back home forgetting everything. One of the participants explained that when he was first diagnosed, on the same day, he was loaded with so much information that he went back home with nothing.

“*When I was diagnosed*, *the nurse advised me on what is diabetes*, *complications*, *foot complications all in one day but I kinda forgot because it was too much to know in one day*, *like I was still trying to come to terms with my sugar being high and on top of that all this information overload came*, *all went from one ear out through the next”*. P14 (35 year old male)

Additionally, another participant stated that he saw all different HCWs in one day with different advice and a lot of information that was too much for him to process in one day.

“*…in one clinic*, *I saw the nurse*, *the doctor*, *the dietician and all advised me on different things and my old brain was full so I forget some of the things they advised”*. P20 (60 year old male)

#### Theme 2: Personal practice and health care practice

Theme 2 has 3 sub-themes which present the perceptions of T2DM participants regarding their behavior and practices relating to their foot care and their perception of services provided at the SOPD clinic and foot care clinic at Rotuma Hospital.

*Fatalistic self—practices*. Two participants mentioned that at home they prefer to walk barefoot so that the soles of their feet are tough and won’t be able to get hurt easily by small rocks and sticks. If they wear shoes all the time, the soles of their feet would become soft and thin and more prone to injuries even by just a small stone.

“*At home*, *I always try to walk barefoot so that my feet can be tough and the skin tough so that I don’t get cuts easily”*. P10 (57 year old female)

Another participant explained that when she feels her legs numb, she knows it’s because of the cold and always soak her feet in warm water.

“*Sometimes when my feet and leg gets numb*, *I always ask my grandchildren to get hot water for me to put my feet inside because that’s the cold that’s making my feet numb”*. P20 (60 year old female)

Another participant explained that her grandmother always tells her that whenever she goes fishing and she gets small cuts on her leg or feet from the coral, she must immediately put kerosene on the cuts to prevent infection.

“*Every time we go fishing in the reefs*, *I always get small cuts from stepping on the corals and my grandmother always tell us to soak a piece of cloth in kerosene and wipe the cuts with it so that’s what I usually do at home when I come back from reef fishing”*. P 26 (58 year old female)

Another participant explained that whenever she washes her feet, she always uses a scrubbing brush and scrubs the soles of her feet:

“*Every time I bath*, *I always hate that hard skin that forms on the soles of my feet so I always use a scrubbing brush to scrub out all those hard skins and sometimes I scrub so hard that blood starts to come out than I stop and wash it with soap”*. P16 (40 year old female),

Two participants shared the influence they get from their close family members who have driven them to do health behavior that is not in-line with the advice from professional HCWs. One of them said that her husband has been advising her to stop taking all the pills from the hospitals as she can get poisoned and take herbal medicine instead.

“*My husband has been telling me to stop taking the tablets from the hospital because it will poison me and is telling me to take the herbal medicine for my diabetes”*. P26 (58 year old female)

*SOPD clinic*. Fifteen participants were satisfied and happy with the services offered to them at the SOPD clinic. They feel welcomed and relaxed whenever they come in for clinic.

“*SOPD clinic is very nice and the staff nurse is always helpful and greets us well*. *There is a relaxed atmosphere around the SOPD clinic”*. P18 (47 year old female)

Another participant highlighted the services provided by health care providers at the SOPD clinic were to her expectations.

“*After the nurse took my blood pressure and checked my sugar*, *my height and weight were also checked and then I was referred to the doctor to see me*. *The doctor greeted me and asked how I was before he advised me on my blood pressure and sugar levels*. *I was also advised on eating healthy foods and take my medicine every day”*. P21 (50 year old female)

However, two participants shared different sentiments to the ones above. One of them said that it is becoming boring for her to go to SOPD clinic every time with the same old routine and nothing exciting and new to see or learn.

“*SOPD clinic is becoming boring for me but I have to because of my diabetes*. *Like every time*, *it’s the same thing over and over again*, *there is no exciting new thing to see or hear when I come for clinic*. *I’ll just come*, *take my pressure and sugar*, *ask about what I ate in the morning and then I wait for the doctor…*.*same routine every time for clinics”*. P19 (63 year old female)

The second participant shared a similar note saying that she feels lazy coming-up for clinics because it’s the same process over and over again.

“*…there’s nothing new is SOPD*, *check the sugar*, *tell me if its high or low*, *ask about what I ate and my medications*, *refill my meds and that’s it… like sometimes I feel lazy coming for clinics because it’s so boring”*. P25 (61 year old female)

*Foot care clinic*. 10 of the participants had never had a foot examination since they were diagnosed with T2DM. From these 10 participants, four have been living with T2DM for more than five years and six participants have been living with T2DM for less than 5 years.

“*I was diagnosed with T2DM 3 year ago and since then*, *I have never had my feet checked by any of the staffs in the hospital”*. P18 (47 year old female)

Seven of the participants, however, did mention that they have had at least one foot examination conducted by the HCW. Two of these participants have been diabetic for less than 5 years and the remaining 5 participants have had diabetes for more than 5 years. One of the participants shared her experience with the foot clinic as satisfactory and pleasant.

“*I have had my feet checked once or twice by the nurse*, *she checked the pulse in my feet and also put some warm and cold water packs and asks me to close my eyes and tell her what I am feeling*. *Then she trimmed off some thick skin around my feet*, *I nearly fell asleep as it felt so good for someone to check and take care of your feet like that*. *Since then*, *I always look forward to my foot clinic days”*. P26 (58 year old female)

In terms of advice given at the foot care clinic, 10 participants mentioned that they have been advised on foot care by the nurse. The advice was mainly of a general nature but no specific advice was given on the steps of washing their feet or foot care. Seven have never received any form of foot care advice from any HCW. One of the 10 participants who received foot care advice explained she was given general advice on foot care.

“*I was advised by 2 staffs on 2 different clinic days about how to look after my feet at home*. *They told me to be careful of my feet*, *to wear shoes all the time and to wash my feet properly”*. P10 (57 year old female)

Similarly, another participant also was given the same advice regarding washing her feet.

“*Yes the staff nurse at the foot clinic advised me to clean my leg and my feet*, *wash it properly every afternoon before going to bed”*. P23 (71 year old female)

*Beneficial self-care practices*. Fifteen participants wash their feet with soap and water every day at home while two of the participants said that they wash whenever they bath but don’t really pay attention to the details of washing their feet. From the 15 participants, four were able to discuss the details of washing their feet. One of the participants explained how she washes her feet at home, which was similar to the other participants’ descriptions.

“*Every time I bath*, *I wet my feet with water then I apply soap and wash it properly*, *in between the toes and then dry it properly and massage my feet with oil but no oil in between the toes*, *it must be dry*, *oil only on the top and the bottom”*. P17 (64 year old female)

Eleven of the participants explained that they wash their feet with soap and water but did not explain further on the specifics of feet washing. All of them had their explanations somewhat similar to what was mentioned by one participant quoted below.

“*Whenever I bath*, *I wash my feet with water and soap and then I dry with a towel”*. P12 (37 year old male)

None of the participants explained that they trim their toe nails at home as part of self-care for their feet.

#### Theme 3: Health seeking behavior

Two sub-themes, namely hospital factors and culture and personal beliefs and traditions are part of theme 3 and they present the perceived influences they have on health seeking behavior of the participants.

*Hospital factors*. Two participants said that they are always embarrassed to come to the hospital because they are always feel shy about holding around their diabetic booklets because everybody will see that they are diabetics. Due to this stigma, they always come to have their diabetic clinics sometimes in the evening. This becomes an issue because the SOPD clinic and foot clinic operates from 8am to 4pm daily.

“*In Rotuma nearly everyone is holding a card or the diabetic booklet and I find it scary and embarrassing and I don’t want to have a card like that so I always dread coming to attend my SOPD clinic with my card”*. P12 (37 year old male)

Another participant also feels the same way about holding her health booklet.

“*Sometimes I just tell the nurse*, *am sorry I forgot my diabetic booklet at home because I am ashamed of holding it around with everybody in the hospital starring”*. P22 (64 year old female)

One participant mentioned that if his daughter, who is a HCW is working, he will always try to delay his coming to the hospital because he knows he will be verbally abused by her for not looking after himself and not following all the advice given from the hospital.

*“…if am sick or I have a cut on my leg and I know my daughter is on duty*, *I’ll try to dodge her and come later to the hospital when she is not at work because she will ask a lot of questions and tell me off as well”*. P13 (58 year old male)

*Culture*, *personal beliefs and traditions*. Four participants said that they prefer to treat their cuts and wounds first with traditional Rotuman medicine and if it fails, then they will go to the hospital. According to these participants, sometimes they use traditional medicine advised by traditional healers in the village and their cuts and wounds heal-up.

“*I remember when I was younger*, *I used to go with my grandmother to get all these leaves and herbs that she always uses for big cuts and wounds that were difficult to heal and she would use it on them*, *sometimes they heal but sometimes they keep on increasing*. *So for me*, *I use them sometimes but when I see after 3 to 4 days that the wound smells*, *I tell the patients please go to the doctor”*. P22 (64 year old female)

Another participant added that he always follows the practices of his parents and grandparents to treat cuts and other sickness first at home and if there was no change, specifically after four days, then he presents to the hospital.

*“I always use leaves and herbal medicine at home first for my cuts and wounds*, *even for my family…not only for cuts and wounds but for other sickness as well*. *But after 4 days*, *if no change then I come up to hospital*. *This was how my parents and even my grandparents use to do with us at home”*. P13 (58 year old male)

Three of the participants shared the same point that if they have a wound on their foot and it is not healing, then they have to go and do some traditional protocols to their chief or leader of the clan to ask for forgiveness as they believe maybe they might have done something to offend the land.

“*I had gone to the bush to feed the pigs and I cut some coconuts from someone else’s land to feed the pigs*. *In the pig pen*, *I stepped on a sharp rock and cut my left foot*. *I applied some leaves and tied a piece of cloth around my foot and came back home*. *I cleaned it with sea water and applied some Rotuman medicine*. *After 3 days*, *my leg was swollen and was hard for me to walk around so the zone nurse came and gave me some antibiotics*. *I also went to our chief and did the traditional thing to ask for forgiveness because I cut the coconuts without asking him first and after a few days*, *my left leg got better”*. P15 (63 year old female)

#### Theme 4: Factors affective footwear practices

Thirteen participants stated they wear shoes whenever they are at home or if they are doing work outside the house or go into the bush. The remaining four participants said that they hardly wear shoes at home and they also shared some of their reasons. This theme has three subthemes which present the perceptions of the participants regarding footwear as a component of foot care.

*Availability and affordability*. The type of shoes commonly worn amongst the 13 participants who stated wearing foot wear was ‘flip-flops’. Flip-flops are available in all the retail shops around the island of Rotuma and are cheap and affordable compared to gum boots, canvas shoes or sandals. One participant explained why she wears flip-flops mostly at home and everywhere she goes:

“*I wear flip-flops because it is the only foot wear available at the post shop and also it is cheap and easy for me to buy*. *I wear it at home*, *when I go with my husband to the garden and other places”*. P10 (57 year old female)

Another participant said that flip flops are cheap and available everywhere in Rotuma shops.

“*Flip flops always*, *everywhere because it is cheap and available in the shop”*. P20 (60 year old female)

Two participants mentioned that they wear closed shoes: either canvas or gum boots whenever they go to the bush,

“*I wear gum boots whenever I go to the bush or to the garden because it is closed and protect my feet well*. *It was bought by my son from Suva and is the only closed shoe I have at home so I always wear it when I go out of the house”*. P15 (63 year old female)

The other participant, on a similar note, said that she always wears canvas shoes for gardening and feeding the pigs.

“*I have a lot of canvas at home sent by my daughter overseas so I use them whenever I go outside for gardening or to go feed the pigs”*. P20 (60 year old female)

On the other hand, one participant mentioned that she has been advised on the appropriate kind of footwear and had had her feet measured but the only challenge is that none of the prescribed footwear is available on the island of Rotuma.

“*…the nurses at the foot care clinic has advised me about the proper foot wear and the kind of foot wear to use but here in Rotuma*, *only flip flops available everywhere and is cheap*, *the gum boots there and the shoes sold at the second hand shop in Motusa but too expensive for me so I just use the flip flop”*. P17 (64 year old female)

*Personal habits*, *societal norms and traditions*. The four participants who said that they hardly wear shoes at home stated that it is normal to be barefoot at home because that was how they were brought-up so they are used to going barefoot at home.

“*I hardly wear shoes at home*, *I only wear shoes if I come up to the government station and as soon as I reach home*, *I take them off and walk around barefoot”*. P14 (35 year old male)

Another reason they hardly wear shoes at home was that they are usually frowned upon by other family members or neighbors if they wear shoes inside the house.

“*You know the mentality in the village*, *when you wear foot wear inside the house they will start to talk about you saying you want to be high shot and be like the tourists so I stopped wearing shoes at home even though I know it is to protect my feet”*. P26 (58 year old female)

*Access to knowledge about appropriate footwear*. Only one of the participants mentioned being advised on told what type of footwear is appropriate for her to wear at home but availability limits her from wearing them. The rest of the 16 participants said that they had never been advised on the type of shoes they should be wearing.

“*All I was told every time was to make sure I wear shoes inside and outside the house*. *The type and the kind of shoes that is right for me*, *no one has told me about them so for me*, *as long as it’s a pair of shoes*, *I wear them”*. P16 (40 year old female)

Another participant explained that he never thought about the type of shoes he wears until he was asked in this interview process as he had never been advised on the type of footwear to use.

*“Now that you asked me in this interview*, *then am thinking that there is a right type of shoes that I should wear*. *I haven’t been advised by any staffs here at the hospital on what kind of shoes is the right one for my feet”*. P13 (58 year old male)

## Discussions

This qualitative study aimed to explore the perceptions of T452DM patients on DFU attending clinics at the SOPD clinic at Rotuma Hospital. The study generated four overarching themes namely; level of knowledge; personal and health care practice; health seeking behavior; and determinants of footwear practices. They highlighted in detail through subthemes the many perceptions T2DM patients have on DFU.

Participants in this study were confused with having high levels of blood glucose and having an unhealthy diet as the basic definition of DM. They were not able to explain the concept of neuropathy and peripheral arterial disease in the development of DFU. Most of them though, were aware that an unhealthy diet and lack of physical activity contributed to DM and DFU. As mentioned by *Coffey et al*., [14], knowledge of DM is important for diabetic patients to first understand before they can understand the foot complications. They also quoted two other studies that were aligned with this point, suggesting general information on DM should first be provided to diabetic patients before focusing on foot care. Another study reported that inadequate levels of knowledge amongst diabetic patients were a major barrier to diabetes and foot self-care [20].

There was a lack of awareness conducted on and education provided for the participants regarding DFU but they do have explanations for foot ulcers that were in-line with their beliefs and traditions. *Coffey et al*., [14] stated in their findings that diabetic patients had alternative perceptions of DFU that may go against medical explanations and as such may greatly influence their health behavior. Poor levels of knowledge and awareness in being able to identify DFU can affect self-foot care; delay the diagnosis of foot ulcers; and cause delays in seeking medical attention that can all eventually lead to increase risks of LLA.

It was also noted that participants in this study lacked essential knowledge in proper foot care as they could not recall being advised about or trained in how to carry out self-foot care practices. A cross-sectional study conducted in India found that those diabetic patients with inadequate knowledge about how to conduct self-foot care tend to develop more DFU [27]. The danger of having inadequate knowledge of foot care is that patients may act like they know everything there is to know and may engage in activities that could put their feet at risk [14]. These discrepancies may indicate that diabetic patients may be engaged in self-care practices that they believe to “know enough” about and were thought to be beneficial when in fact it could be that they are harmful and possibly fatalistic practices that are not in-line with current DM care or foot care guidelines [20].

The cornerstone to protect the feet from DFU foot complications is through the motivation and empowerment of diabetic patients to take care of themselves and their feet. Such can only be achieved through awareness and education to improve levels of knowledge. So far this has been the most crucial tool for prevention of DFU [27–30].

Many factors were also found to have influenced the Health Seeking Behavior of participants. Stigma associated with the personal health booklets for diabetes records was one of the main factors of influence. Other factors included having relatives as HCWs in the hospital as well as culture and traditions through the use of traditional healers and traditional medicine in the communities. Several studies have acknowledged the impact of culture and personal beliefs on diabetic patients HSB. These beliefs and traditions could delay their presentation to the hospital for medical assistance. Regardless of the number of education and awareness sessions conducted around safe foot care practices, diabetic patients will ignore this if it is contrary to their personal beliefs and culture [17, 31–34]. *Sayampanathan et al*., [16] emphasized that when compared with knowledge status, patient’s personal belief system has a bigger role in influencing foot care practices amongst diabetic patients.

Footwear practices were also highlighted in the study with participants wearing some form of footwear at home, either inside or outside. However, the majority of participants still lacked the knowledge about which is considered appropriate to wear. *van Netten et al*., 2018 affirmed that inappropriate footwear or no footwear at all causes more mechanical stress to the feet which further increases the likelihood of developing DFU. Two other studies reported the same association between wearing inappropriate footwear and DFU [29, 35].

Factors influencing the behavior of participants regarding wearing proper footwear included lack of advice from health professionals on which footwear is appropriate. Several studies agreed with this finding that HCWs hardly advise on the appropriate footwear to be worn by diabetic patients [13, 36–38].

In addition, prescribed footwear is not available and usually expensive. This finding is consistent with several international studies highlighting that most diabetic patients, even those who are aware of the type of footwear to use, are unable to because they are expensive to buy [13, 36, 38]. A study conducted in India concurred with the findings of this study, mentioning that prescribed footwear is usually not available in shops all over India [39].

Cultures, traditions and societal norms also prevented participants from wearing foot wear at home. Several international studies also reported the same findings that even though they have been advised on the benefits of wearing footwear, messages as such are ignored due to personal practices that have been observed in their houses for generations [13, 17, 37]. In Africa, barefoot is a common societal norm [39].

The findings of this study has highlighted many gaps in the knowledge of DM, DFU and foot care and have also identified areas of practice that needed most improvements to promote foot care and prevent of DFU. More emphasis to be placed on foot care advice to diabetic patients. Foot care advice must be given to all diabetic patients who haven’t had DFU to promote prevention.

### Limitations

Despite data saturation being reached, the study has a small sample size. Rotuma hospital is the only hospital on the island where the study was done. Areas with more than one hospital may generate different perceptions to this study. The study was conducted in a rural area in a maritime zone and the perceptions of those of the mainland may differ therefore, the results may not be generalized to those outside of the study population.

## Conclusion

In conclusion, T62DM patients had poor practical knowledge about DFU and foot care practices than that is beneficial to their feet. This has been attributed to lack of advice from health professionals; personal belief states; culture and societal norms; lack of resources; stigmatization; and the lack of engagement with health professionals on foot care. Prevention of DFU can be achieved through intensive repetitive awareness and education sessions with the participants so that they are able to differentiate perceived knowledge from fact regarding DFU and foot care. Perceptions of patients must always be considered when formulating prevention strategies regarding DFU, foot care and footwear practices. It is needed to conduct another study to explore the perceptions of family members and caregivers at home. It also will be helpful to explore the impact of SNAPs clinic on foot self-care practices is also recommended in future research.
